# Inflammatory Bowel Disease Is an Independent Risk Factor for Metabolic Dysfunction–Associated Steatotic Liver Disease in Lean Individuals

**DOI:** 10.1093/ibd/izad175

**Published:** 2023-08-22

**Authors:** Samuel J Martínez-Domínguez, Sandra García-Mateo, Carla J Gargallo-Puyuelo, Beatriz Gallego-Llera, Pilar Callau, Carolina Mendi, María Teresa Arroyo-Villarino, Miguel Ángel Simón-Marco, Javier Ampuero, Fernando Gomollón

**Affiliations:** Department of Gastroenterology, Lozano Blesa University Hospital, Zaragoza, Spain; Digestive Pathology Translational Research Group, Aragón Health Research Institute, Zaragoza, Spain; Department of Medicine, School of Medicine, University of Zaragoza, Zaragoza, Spain; Department of Gastroenterology, Lozano Blesa University Hospital, Zaragoza, Spain; Digestive Pathology Translational Research Group, Aragón Health Research Institute, Zaragoza, Spain; Department of Medicine, School of Medicine, University of Zaragoza, Zaragoza, Spain; Department of Gastroenterology, Lozano Blesa University Hospital, Zaragoza, Spain; Digestive Pathology Translational Research Group, Aragón Health Research Institute, Zaragoza, Spain; Department of Medicine, School of Medicine, University of Zaragoza, Zaragoza, Spain; Digestive Pathology Translational Research Group, Aragón Health Research Institute, Zaragoza, Spain; Primary care center Delicias Sur, Zaragoza, Spain; Primary care center Universitas, Zaragoza, Spain; Department of Gastroenterology, Lozano Blesa University Hospital, Zaragoza, Spain; Digestive Pathology Translational Research Group, Aragón Health Research Institute, Zaragoza, Spain; Department of Medicine, School of Medicine, University of Zaragoza, Zaragoza, Spain; Department of Gastroenterology, Lozano Blesa University Hospital, Zaragoza, Spain; Digestive Pathology Translational Research Group, Aragón Health Research Institute, Zaragoza, Spain; Department of Medicine, School of Medicine, University of Zaragoza, Zaragoza, Spain; Department of Digestive Diseases, Virgen del Rocío University Hospital, Sevilla, Spain; Department of Medicine, University of Sevilla, Sevilla, Spain; Clinical and Translational Research Group in Liver and Digestive Diseases, Biomedicine Institute of Sevilla, Sevilla, Spain; Centro de Investigación Biomédica en Red de Enfermedades Hepáticas y Digestivas, Madrid, Spain; Department of Gastroenterology, Lozano Blesa University Hospital, Zaragoza, Spain; Digestive Pathology Translational Research Group, Aragón Health Research Institute, Zaragoza, Spain; Department of Medicine, School of Medicine, University of Zaragoza, Zaragoza, Spain; Centro de Investigación Biomédica en Red de Enfermedades Hepáticas y Digestivas, Madrid, Spain

**Keywords:** lean MASLD, liver fibrosis, inflammatory bowel disease, Crohn’s disease, ulcerative colitis

## Abstract

**Background:**

Despite classical association between metabolic dysfunction–associated steatotic liver disease (MASLD) and obesity, there is increasing evidence on the development of MASLD in lean individuals. The aim of the study was to assess the prevalence and risk factors of MASLD and significant liver fibrosis in lean participants with inflammatory bowel disease (IBD).

**Methods:**

This was a cross-sectional, case-control study including 300 lean cases with IBD and 80 lean controls without IBD, matched by sex and age. All participants underwent a liver ultrasound, transient elastography, and laboratory tests.

**Results:**

The lean IBD group showed a significantly higher prevalence of MASLD compared with lean non-IBD group (21.3% vs 10%; *P =* .022), but no differences were observed in the prevalence of significant liver fibrosis (4.7% vs 0.0%; *P* = 1.000). No differences were found between the prevalence of MASLD in IBD and non-IBD participants who were overweight/obese (66.8% vs 70.8%; *P =* .442). In addition, the prevalence of MASLD was significantly higher in the overweight/obese IBD group compared with the lean IBD group (*P <* .001). IBD was an independent risk factor for MASLD in lean participants (odds ratio [OR], 2.71; 95% confidence interval [CI], 1.05-7.01; *P =* .04), after adjusting for classic metabolic risk factors and prior history of systemic steroid use. Nevertheless, no association between IBD related factors and MASLD was identified in lean IBD participants. When the overweight/obese and lean IBD groups with MASLD were compared, the overweight/obese IBD group with MASLD showed higher levels of the homeostatic model assessment of insulin resistance (OR, 1.49; 95% CI, 1.11-1.98; *P =* .007) and history of smoking (OR, 4.66; 95% CI, 1.17-18.49; *P =* .029).

**Conclusions:**

MASLD prevalence was higher in the lean IBD group compared with lean non-IBD group, independent of classic metabolic risk factors.

## Graphical Abstract

**Graphical Abstract:**
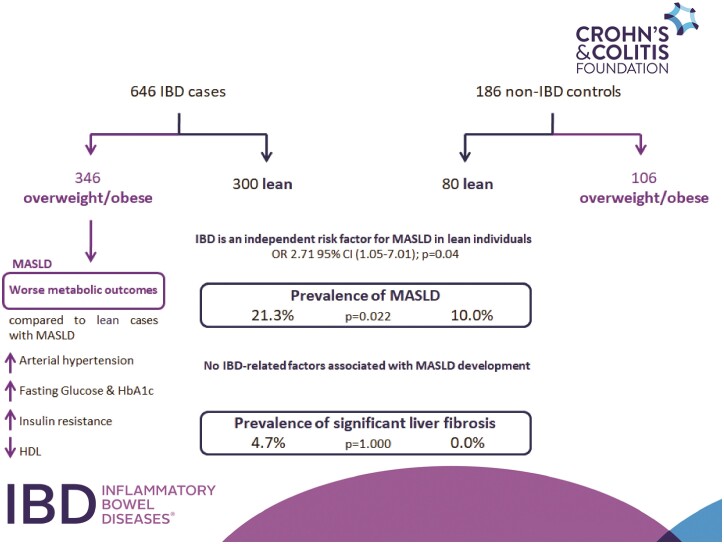

**Key Messages**

- What is already known? Obesity is a well-stablished risk factor for metabolic dysfunction–associated steatotic liver disease (MASLD), but not all patients with MASLD in the general population are overweight/obese.
- What is new here? Inflammatory bowel disease (IBD) itself was a risk factor for MASLD in lean individuals, independent of classic metabolic risk factors and prior history of systemic steroid use. No association between IBD-related factors and MASLD was identified in the lean IBD group.
- How can this study help patient care? These findings suggest that MASLD should also be suspected in lean patients with IBD, and lifestyle measures could be prescribed to treat or prevent this entity.

## Introduction

The prevalence of metabolic dysfunction–associated steatotic liver disease (MASLD) is increasing significantly over time, affecting up to a third of the world’s population.^1^ Thus, MASLD has become a leading cause of liver disease and transplantation.^2^ It comprises a wide histological spectrum, commonly nonalcoholic fatty liver (liver steatosis affecting ≥5% hepatocytes with no evidence of inflammation), and less frequently, it progresses to nonalcoholic steatohepatitis (liver steatosis accompanied by inflammation and hepatocyte injury) and liver fibrosis.^3^ The development of fibrosis stages F3 or F4 has been associated with an increased risk of decompensate cirrhosis, hepatocellular carcinoma, and death.^4^ In fact, MASLD was recently identified as the fastest growing cause of hepatocellular carcinoma in liver transplant candidates.^5^

Although the pathogenesis of MASLD is not fully understood, many pathophysiological links with metabolic syndrome have been reported. Indeed, the liver is considered a key determinant of metabolic abnormalities.^6^ MASLD is associated with visceral adiposity, insulin resistance, and atherogenic dyslipidemia, conditions closely related to increased cardiovascular risk. However, MASLD is a well-recognized independent risk factor for atherosclerotic cardiovascular disease, regardless of associated diseases.^6-8^

Despite classical association between MASLD and obesity, there is cumulative evidence that not all overweight/obese individuals develop MASLD and that not all patients with MASLD are necessarily obese.^9^ The term “lean MASLD” is used when it develops in underweight or normal-weight individuals based on body mass index (BMI). It was first reported in Asia, and its prevalence is around a quarter of individuals with MASLD.^10^ Noteworthy, lean patients with MASLD have worse long-term outcomes than lean patients without MASLD, suggesting that BMI does not adequately represent metabolic risk of these individuals.^11^

Although inflammatory bowel diseases (IBDs) are chronic inflammatory entities mainly involving the gastrointestinal tract, liver extraintestinal manifestations often coexist. Several factors such as lifestyle, increasing age, and the development of new therapeutic targets have probably changed the nutritional status of IBD patients, and currently being overweight and obese comprises the most frequent nutritional disorders.^12,13^

Previous studies reported a prevalence of MASLD in IBD population higher than 40%, noting that MASLD is a major problem in patients with IBD.^14^ In addition, IBD-related risk factors have been independently associated with MASLD and liver fibrosis, the determinant long-term prognostic marker of liver disease. Nevertheless, to the best of our knowledge, the evidence regarding MASLD and liver fibrosis in lean population with IBD is scarce.

Therefore, the primary aim of this study was to assess the prevalence and risk factors for MASLD and significant liver fibrosis in lean individuals with IBD.

## Methods

### Study Design

Cross-sectional, case-control study performed between October 2020 and October 2021 in the IBD outpatient unit of Lozano Blesa University Hospital (Zaragoza, Spain). This study focused in particular on lean participants from a larger cohort of IBD patients and healthy control subjects without IBD.

Consecutive outpatients from our IBD unit with an established diagnosis of ulcerative colitis (UC), Crohn’s disease (CD), or indeterminate colitis were enrolled in the cases group. Control subjects were consecutive outpatients without IBD recruited in 2 primary care centers belonging to the same health area. Cases and controls were matched by sex and age. The following exclusion criteria were applied for both cases and controls: significant alcohol consumption (>20 g/d for women and >30 g/d for men), current or previous treatment with methotrexate and/or systemic steroids in the last 2 years, pregnancy, refusal to participate, or previous diagnosis of chronic liver disease different from nonalcoholic fatty liver disease such as viral, genetic (Wilson, alpha-1 antitrypsin deficiency, hemochromatosis, or storage diseases), and immune mediated. All participants included in the study signed the informed written consent.

All participants were interviewed, their medical records were reviewed, anthropometric measurements and a fasting blood sample were taken, and liver steatosis and fibrosis were assessed by noninvasive methods.

We collected the following demographic variables through clinical interview and medical record review: age, sex, smoking habit, alcohol consumption, and comorbidities (arterial hypertension, type 2 diabetes mellitus [DM], lipid-lowering treatment, chronic kidney disease, cardiovascular disease, and cerebrovascular disease). In addition, we collected IBD-related features: date of diagnosis, type of IBD, location, pattern (CD), perianal disease, extraintestinal disease, previous surgeries, and current and previous treatment. After the clinical interview, we measured the following anthropometric variables: weight, height, and perimeters (arm, waist, and hip). From these measurements, body mass index (BMI), waist-to-hip index, and body fat percentage were calculated. Finally, a fasting blood sample was drawn to determine fasting glucose, glycosylated hemoglobin, fasting insulin, albumin, creatinine, cholesterol (total, low-density lipoprotein, and high-density lipoprotein), triglycerides, liver profile, C-reactive protein, blood count, coagulation, hepatitis C virus antibody, hepatitis B surface antigen, and anti-hepatitis B core antigen.

### Diagnostic Criteria and Definitions

Patients were classified according to BMI status in the following groups: underweight (<18.5 kg/m^2^), normal weight (18.5-24.9 kg/m^2^), overweight (25.0-29.9 kg/m^2^), and obese (≥30 kg/m^2^). For non-Asian participants, lean individuals were defined as BMI <25 kg/m^2^.

IBD diagnosis was established according to the European Crohn’s and Colitis Organisation criteria.^14,15^ Clinical remission was defined as partial Mayo score ≤1 (UC) or Harvey-Bradshaw Index ≤4 points (CD). Localization and extension of IBD were defined using the Montreal classification for CD (ileal, colonic, ileocolonic, isolated upper disease) or UC (proctitis, left sided, pancolitis).

After ≥4 hours of fasting, both cases and controls underwent liver ultrasound (US) and transient elastography (TE) by 2 trained gastroenterologists. For abdominal B-mode US, a low-frequency convex probe was used, and for TE, a FibroScan 430 Mini (Echosens) was used. Ultrasonographic diagnosis of steatosis was graded as follows: mild in case of a slight increase of liver echogenicity (compared with right kidney parenchyma) and normal visualization of portal vein wall and diaphragm, moderate when there was a higher increase of liver echogenicity with slight impaired of portal vein wall and diaphragm, and severe in case of marked increase of liver echogenicity with absence or poor visualization of portal vein wall, diaphragm, or posterior right liver lobe.^16^

MASLD diagnosis was established in case of any grade of US steatosis or controlled attenuation parameter >248 dB/m. Significant liver fibrosis was considered in case of liver stiffness ≥8 kPa, as this cutoff has demonstrated to accurately rule out advanced liver fibrosis.^17,18^ TE values were only considered valid in participants with at least 10 measurements and interquartile range/median liver stiffness ratio ≤30%.

### Study Endpoints

The primary outcomes of the study were (1) to compare the prevalence of MASLD between the lean IBD group and lean non-IBD group and (2) to assess the prevalence of significant liver fibrosis in the lean IBD group and lean non-IBD group.

The secondary outcomes of the study were (1) to identify risk factors associated with MASLD in lean individuals, especially IBD-related risk factors; and (2) to compare MASLD prevalence, significant liver fibrosis prevalence, and metabolic profile between the lean IBD group and overweight/obese IBD group.

### Statistical Analysis and Ethical Statement

Qualitative variables were presented as absolute and relative frequencies. For quantitative variables, normality was assessed using Kolmogorov-Smirnov test, in which *P* ≥ .05 means normality. Quantitative variables were presented as mean ± SD if they followed normal distribution, and in case of non-normal distribution, median and range were used. The relationship between categorical variables was assessed using the chi-square test or Fisher test, as appropriate. The Student *t* test or Mann-Whitney *U* test was used for quantitative variables, as appropriate. In addition, a logistic regression multivariable analysis was performed, and data presented as adjusted odds ratio (OR) and a 95% confidence interval (CI). Multivariable analysis included variables found to be significant in univariable analysis and biologically relevant variables such as age, sex, BMI, waist-to-hip ratio, arterial hypertension, type 2 DM, insulin resistance, smoking habit, lipid-lowering drugs, and previous steroid use. A *P* value <.05 was considered as statistically significant. Jamovi version 2.3.21 (https://www.jamovi.org/) was used to carry out the statistical analysis.

The sample size was estimated according to the expected number of IBD patients seen in our outpatient unit during 1 year. The case-control ratio was 3:1, and they were matched by age and sex.

The study was performed in agreement with the Helsinki declaration and was approved by Aragón Ethics Committee (code PI19/451).

## Results

### Baseline Characteristics of Participants

**Figure 1.**
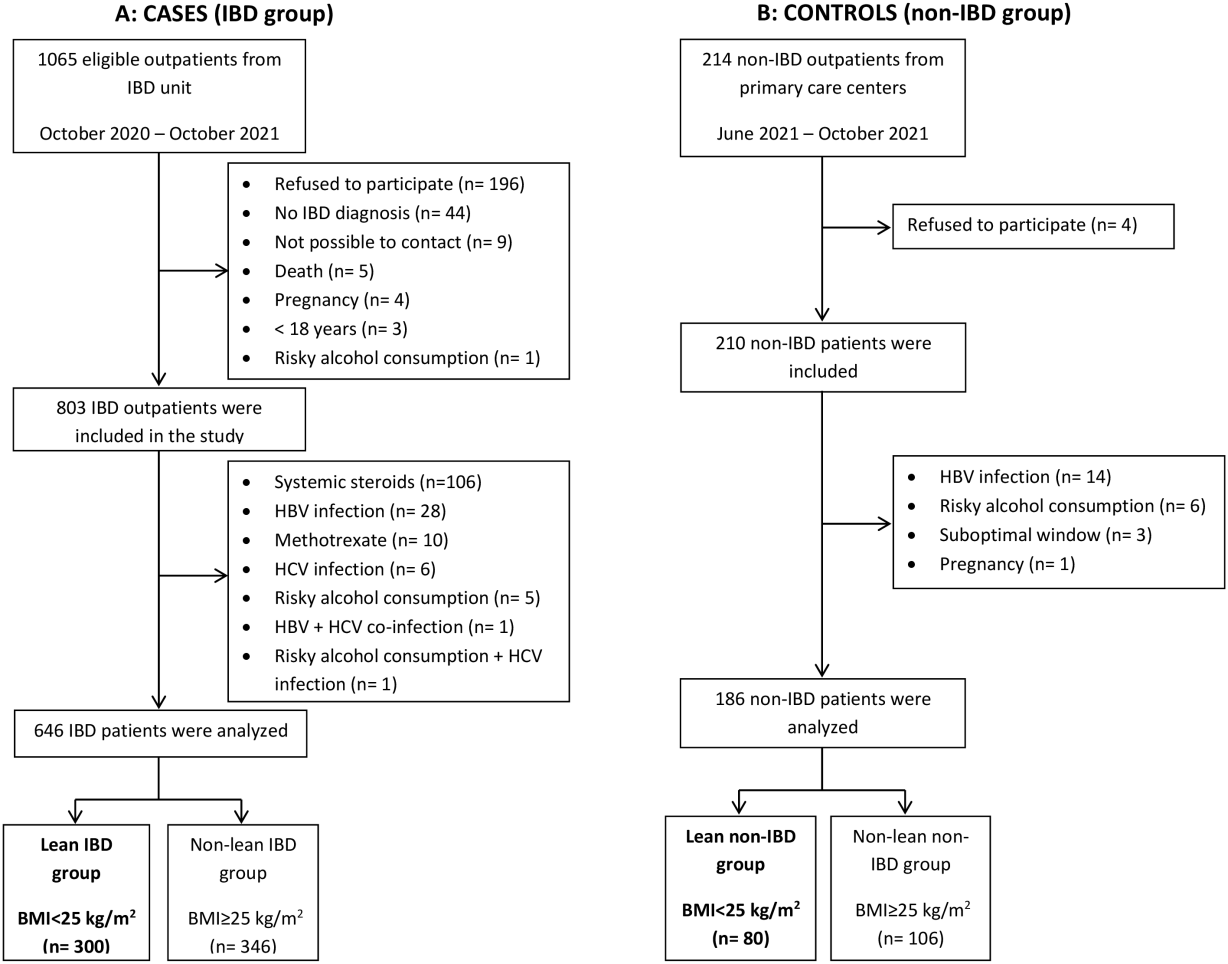
Flowchart of participant selection: (A) cases, (B) controls. BMI, body mass index; HBV, hepatitis B virus; HCV, hepatitis C virus; IBD, inflammatory bowel disease.

A total of 1065 outpatients were visited in our IBD unit during 1 year, and among them 803 agreed to participate in the study. After applying the inclusion and exclusion criteria, 300 lean IBD and 346 overweight/obese IBD participants were analyzed. Regarding control subjects, 210 coming from primary care centers of the same health area were included. After checking inclusion and exclusion criteria, 80 lean non-IBD and 106 overweight/obese non-IBD participants were analyzed (**Figure 1**).

Due to the study design, no differences in age and sex were found between the IBD and non-IBD groups. In addition, the presence of type 2 DM, arterial hypertension, active smoking habit, and cardiovascular and cerebrovascular disease was homogeneous between both groups (**Table 1**). Participants came from Europe, Africa, and South America. Despite a similar BMI (median 22.5 [range, 15.4-25.0] kg/m^2^ vs 22.4 [range, 16.7-24.8] kg/m^2^; *P =* .788) and body fat percentage (median 27.7% [range, 6.4%-39.8%] vs 26.7% [range, 7.8%-38.8%]; *P =* .413) in the lean IBD group and lean non-IBD group, and the lean IBD group showed a significantly higher waist-to-hip ratio (0.86 ± 0.08 vs 0.84 ± 0.08; *P =* .038) and a significantly higher prevalence of MASLD (21.3% vs 10.0%; *P =* .022). Regarding laboratory tests, the lean non-IBD group had worse lipid profile and lower use of lipid-lowering drugs. No differences in glycemic control, insulin resistance, and liver profile were found.

**Table 1.** Baseline characteristics of the lean IBD group and lean non-IBD group.

Characteristic	Lean IBD group (n = 300)	Lean non-IBD group (n = 80)	*P* value^a^
Clinical			
Female	176 (58.7)	46 (57.5)	.851
Age, y	46.8 ± 13.1	44.1 ± 15.5	.118
Active smokers	63 (21.0)	15 (18.8)	.658
Previous smokers	121 (40.3)	22 (27.5)	.035^b^
Passive smokers	33 (11.0)	10 (12.5)	.707
Illegal drug consumption	1 (0.3)	0 (0.0)	1.000
Chronic kidney disease	23 (7.7)	15 (18.8)	.003^b^
Cardiovascular disease	6 (2.0)	0 (0.0)	.350
Cerebrovascular disease	2 (0.7)	2 (2.5)	.196
Arterial hypertension	26 (8.7)	8 (10.0)	.710
Type 2 DM	12 (4.0)	3 (3.8)	1.000
Hypercholesterolemia drugs	35 (11.7)	3 (3.8)	.036^b^
Triglyceride-lowering drugs	10 (3.3)	2 (2.5)	1.000
Lean MASLD	64 (21.3)	8 (10.0)	.022^b^
Significant liver fibrosis in patients with lean MASLD (n = 72)	3 (4.7)	0 (0.0)	1.000
Anthropometric measures			
BMI, kg/m^2^	22.5 (15.4-25.0)	22.4 (16.7-24.8)	.788
Waist-hip ratio, mean ± SD	0.86 ± 0.08	0.84 ± 0.08	.038^b^
Body fat percentage (CUN-BAE), %	27.7 (6.4-39.8)	26.7 (7.8-38.8)	.413
Body fat percentage (BAI), %	26.2 ± 3.7	26.6 ± 4.1	.418
Laboratory tests			
Fasting glucose levels, mg/dL	92.0 (67.0-299.0)	93.0 (76.0-146.0)	.364
HbA1c, %	5.4 (4.4-12.8)	5.4 (4.2-8.9)	.143
Creatinine, mg/dL	0.80 (0.37-4.10)	0.82 (0.58-1.27)	.463
Serum albumin, g/dL	4.05 (0.98-5.00)	4.04 (3.46-4.80)	.389
Total cholesterol, mg/dL	190.0 ± 40.3	197.0 ± 36.1	.169
LDL, mg/dL	107.0 ± 33.9	115.0 ± 34.3	.089
HDL, mg/dL	64.1 ± 17.7	63.5 ± 14.0	.780
Triglycerides, mg/dL	80 (29-3087)	81 (37-313)	.962
Total bilirubin, mg/dL	0.48 (0.13-2.80)	0.45 (0.13-1.88)	.211
AST, UI/L	20 (10-139)	19 (10-36)	.619
ALT, UI/L	15 (5-131)	16 (5-55)	.687
GGT, UI/L	16 (6-343)	15 (5-97)	.760
ALP, UI/L	67 (28-241)	64 (31-155)	.985
C-reactive protein, mg/L	1.75 (0.02-219.00)	0.87 (0.00-23.20)	<.001^b^
Fasting insulin levels, µU/mL	5.06 (2.00-65.00)	6.23 (2.00-46.90)	.269
HOMA-IR	1.13 (0.19-24.4)	1.44 (0.39-10.5)	.223

Values are n (%), mean ± SD, or median (range). Lean refers to a BMI <25 kg/m^2^.

Abbreviations: ALP, alkaline phosphatase; ALT, alanine aminotransferase; AST, aspartate aminotransferase. BAI, Body Adiposity Index; BMI, body mass index; CUN-BAE, Clínica Universidad de Navarra-Body Adiposity Estimator; DM, diabetes mellitus; GGT, gamma-glutamyl transferase; HbA1c, glycosylated hemoglobin; HDL, high-density lipoprotein; HOMA-IR, homeostatic model assessment of insulin resistance; IBD, inflammatory bowel disease; LDL, low-density lipoprotein; MASLD, metabolic dysfunction–associated steatotic liver disease.

^a^For the qualitative variables, chi-square test or Fisher’s test was used, and for quantitative variables Student *t* test or Mann-Whitney *U* test was used, as appropriate.

^b^Statistical significance: *P <* .05.

The study included 157 (52.3%) patients with UC, 137 (45.7%) with CD, and 6 (2.0%) with indeterminate colitis. The median age at IBD diagnosis was 31.5 (range, 9.0-71.0) years and the median IBD duration was 12.0 (range, 0.0-46.0) years. Although patients receiving systemic steroids currently or in the previous 2 years were excluded, 82 (27.4%) had previously received them in the last 5 years and 196 (65.6%) had received them since diagnosis. No patient received methotrexate. IBD-related features are summarized in **Table 2**.

**Table 2.** IBD-related features of lean cases.

Characteristic	Lean IBD group (n = 300)
Age at diagnosis, y	31.5 (9.0-71.0)
IBD duration, y	12.0 (0.0-46.0)
**Type of IBD**	
UC	157 (52.3)
CD	137 (45.7)
IC	6 (2.0)
**Extent of UC** (n = 157)	
Proctitis	41 (26.1)
Left-sided colitis	53 (33.8)
Extensive colitis	63 (40.1)
**Extent of CD** (n = 137)	
Ilecolonic (L3)	64 (46.7)
Terminal ileum (L1)	47 (34.3)
Colonic (L2)	15 (10.9)
Ileocolonic (L3) + L4	4 (2.9)
Isolated upper disease (L4)	4 (2.9)
Terminal ileum (L1) + L4	2 (1.5)
Colonic (L2) + L4	1 (0.7)
**CD behavior** (n = 137)	
Inflammatory	87 (63.5)
Stricturing	35 (25.5)
Penetrating	14 (10.2)
Stricturing + penetrating	1 (0.7)
**Previous surgery**	
Bowel resection	47 (15.7)
Perianal	13 (4.3)
**Extraintestinal manifestations**	
Arthropathy	37 (12.3)
Arthropathy + skin	15 (5)
Skin	9 (3.0)
Ocular	2 (0.7)
Arthropathy + skin + ocular	1 (0.3)
Perianal disease	40 (13.3)
IBDQ-9	51 (27-59)
**Clinical severity**	
**Partial Mayo score** (n = 157)	
Remission	151 (96.2)
Active disease	6 (3.8)
**Harvey-Bradshaw index** (n = 137)	
Remission	130 (94.9)
Active disease	7 (5.1)
**Current treatment** ^a^	
Only 5-ASA	107 (35.7)
Anti-TNF	65 (21.7)
No treatment	50 (16.7)
Only Thiopurines	29 (9.7)
Ustekinumab	17 (5.7)
Biologic therapy + thiopurines	15 (4.9)
Vedolizumab	8 (2.7)
Tofacitinib	3 (1.0)
Other	6 (2.0)
**Previous treatment** ^a^	
Systemic steroids since diagnosis	196 (65.6)
Systemic steroids last 5 y	82 (27.4)
Other biologic agent	62 (20.7)

Values are median (range) or n (%).

Abbreviations: 5-ASA, 5-aminosalicylic acid; CD, Crohn’s disease; IBD, inflammatory bowel disease; IBDQ-9, 9-item IBD, Inflammatory Bowel Disease Questionnaire; IC, indeterminate colitis; TNF, tumor necrosis factor; UC, ulcerative colitis.

^a^No patient received systemic steroids or methotrexate at inclusion or in the previous 2 years.

### Higher Prevalence of MASLD in the Lean IBD Group Compared With the Lean Non-IBD Group

**Figure 2.**
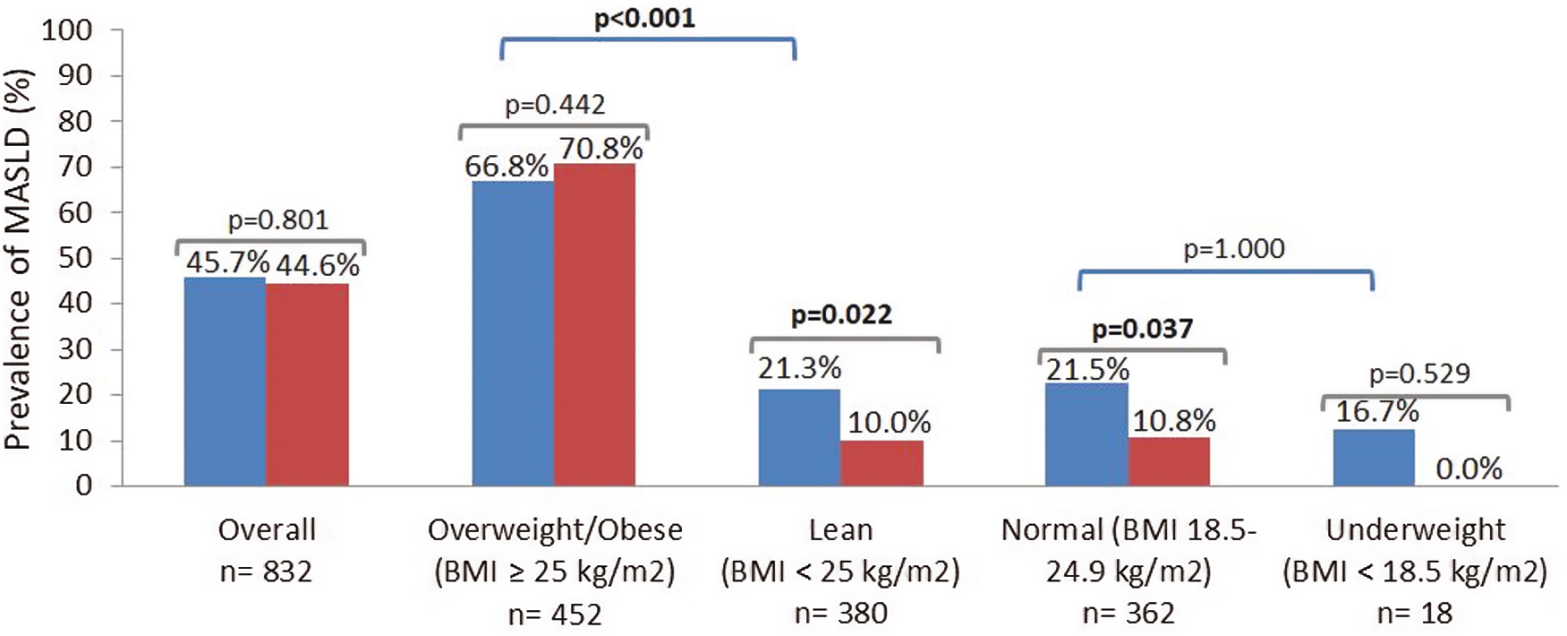
Prevalence of metabolic dysfunction–associated steatotic liver disease (MASLD) in cases and controls according to body mass index (BMI) status. Blue bars: cases (inflammatory bowel disease). Red bars: controls (non–inflammatory bowel disease). *P* values in bold indicate statistical significance (*P <* .05).

When MASLD prevalence was analyzed according to BMI status (**Figure 2**), we noticed higher prevalence in overweight/obese participants (66.8% in the IBD group and 70.8% in the non-IBD group) compared with lean participants (21.3% in the IBD group and 10.0% in the non-IBD group). MASLD prevalence in the IBD and non-IBD groups was similar when all participants (45.7% in the IBD group vs 44.6% in the non-IBD group; *P =* .801) and overweight/obese participants (66.8% in the IBD group vs 70.8% in the non-IBD group; *P =* .442) were analyzed. Nevertheless, a significantly higher prevalence of MASLD was found in the lean IBD group (21.3% in the IBD group vs 10.0% in the non-IBD group; *P =* .022) and normal weight IBD group (21.5% in the IBD group vs 10.8% in the non-IBD group; *P =* .037) compared with their respective control subjects. While MASLD prevalence was significantly higher in the overweight/obese IBD group compared with the lean IBD group (66.8% vs 21.3%; *P <* .001), no statistically significant differences were found between the IBD group with normal and low BMI (21.5% vs 16.7%; *P* = 1.000).

**Figure 3.**
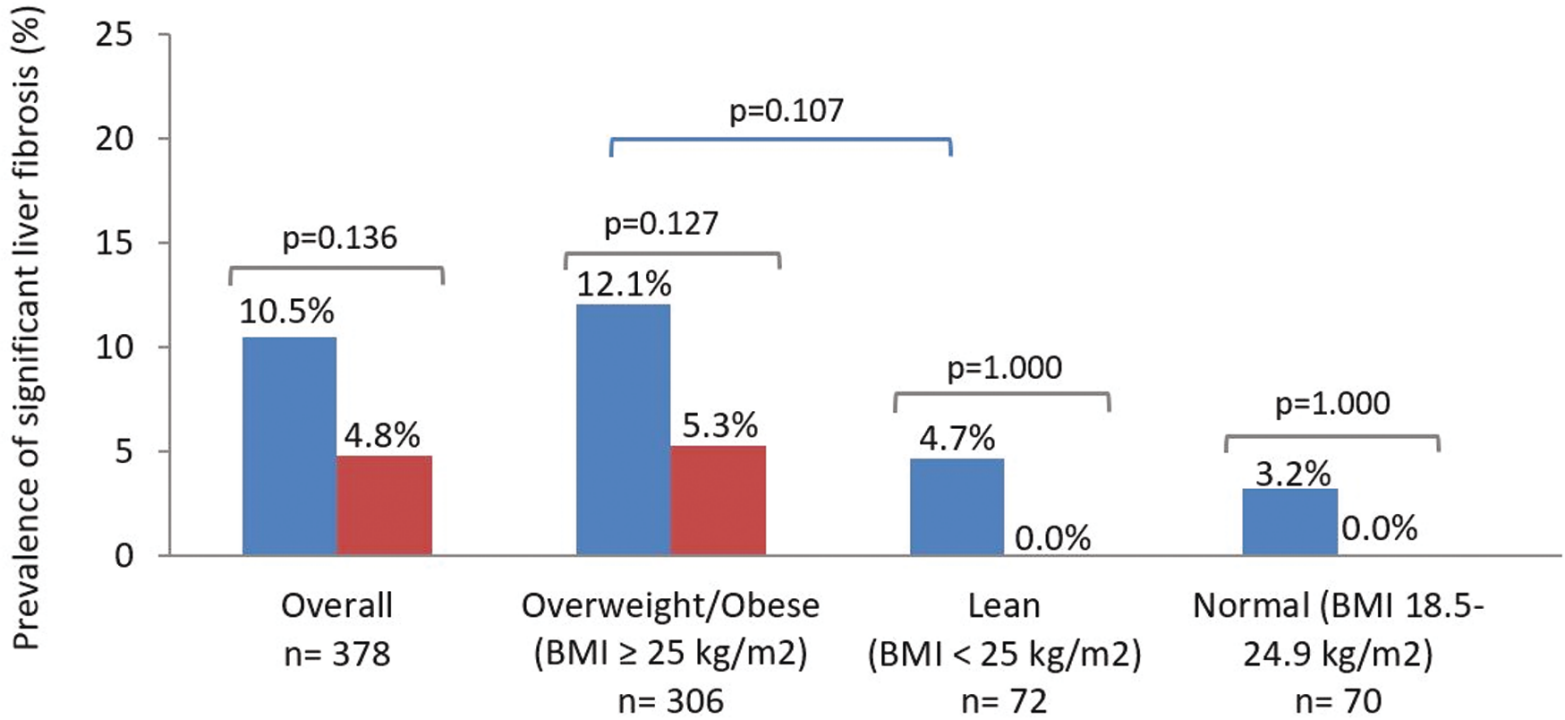
Prevalence of significant liver fibrosis in participants with metabolic dysfunction–associated steatotic liver disease according to body mass index (BMI) status. Blue bars: cases (inflammatory bowel disease). Red bars: controls (non-IBD).

We observed no statistically significant differences between the prevalence of significant liver fibrosis in the lean IBD group and the lean non-IBD group (4.7% in cases vs 0.0% in controls; *P* = 1.000) (**Figure 3**). Moreover, no differences were observed when significant liver fibrosis was analyzed in the overweight/obese and normal-weight groups.

Although univariable analysis showed significantly lower total cholesterol, low-density lipoprotein, and high-density lipoprotein levels in the lean IBD group with disease activity compared with those in remission, these findings were not confirmed in multivariable analysis. In addition, the analysis according to IBD activity found no significant differences in the prevalence of significant liver fibrosis. However, liver stiffness levels were significantly higher in lean patients with IBD activity compared with those in remission (OR, 1.27; 95% CI, 1.06-1.52; *P =* .011) (**Supplementary Table 1**).

### IBD Is an Independent Risk Factor for MASLD in Lean Participants

**Figure 4.**
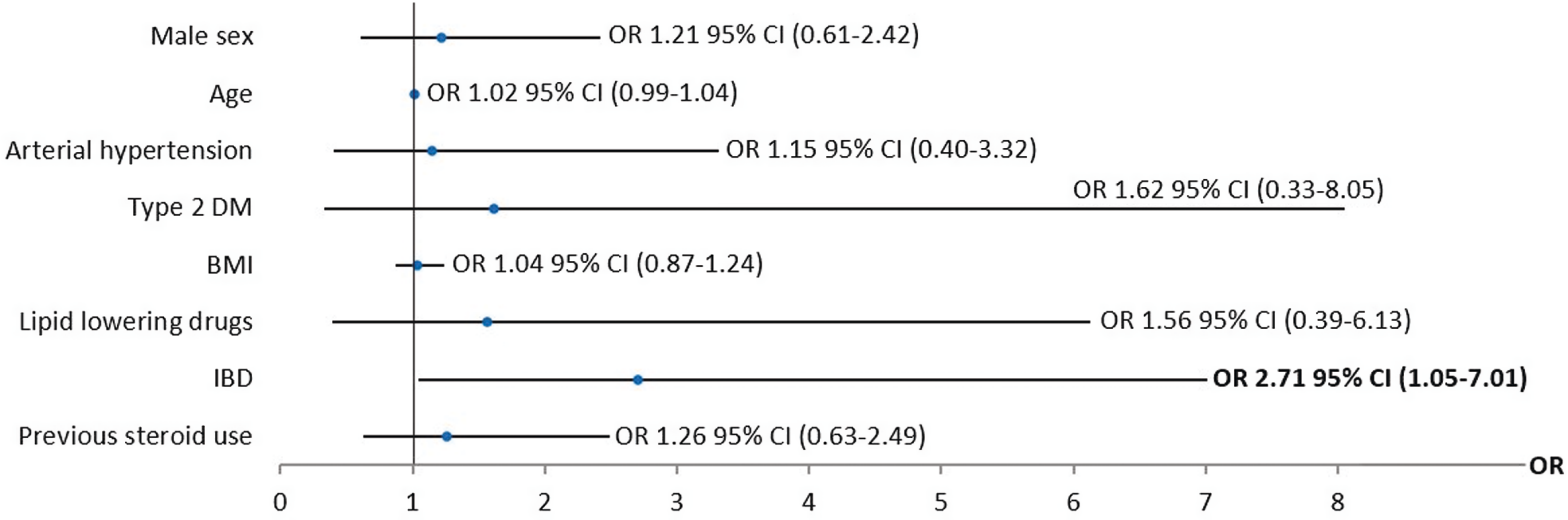
Logistic regression analysis of metabolic dysfunction–associated steatotic liver disease risk factors in lean participants. Data are presented as odds ratio (OR) and 95% confidence interval (CI). Data were adjusted by sex, age, body mass index (BMI), waist-to-hip ratio, arterial hypertension, type 2 diabetes mellitus (DM), insulin resistance, smoking habit, lipid-lowering drugs use, and previous steroid use. IBD, inflammatory bowel disease.

When all lean participants (IBD and non-IBD) were analyzed, MASLD patients showed an increased BMI and anthropometric measures, poorer glycemic and lipid control, and increased insulin resistance in univariable analysis (**Supplementary Table 2**). Multivariable analysis confirmed that IBD was an independent risk factor for MASLD in lean participants (OR, 2.71; 95% CI, 1.05-7.01; *P =* .04) after adjusting for age, sex, arterial hypertension, type 2 DM, insulin resistance, hyperlipidemia, BMI, waist-to-hip ratio, smoking habit, and previous steroid use (**Figure 4**).

### No Association Between IBD-Related Factors and MASLD in Lean IBD Participants

**Figure 5.**
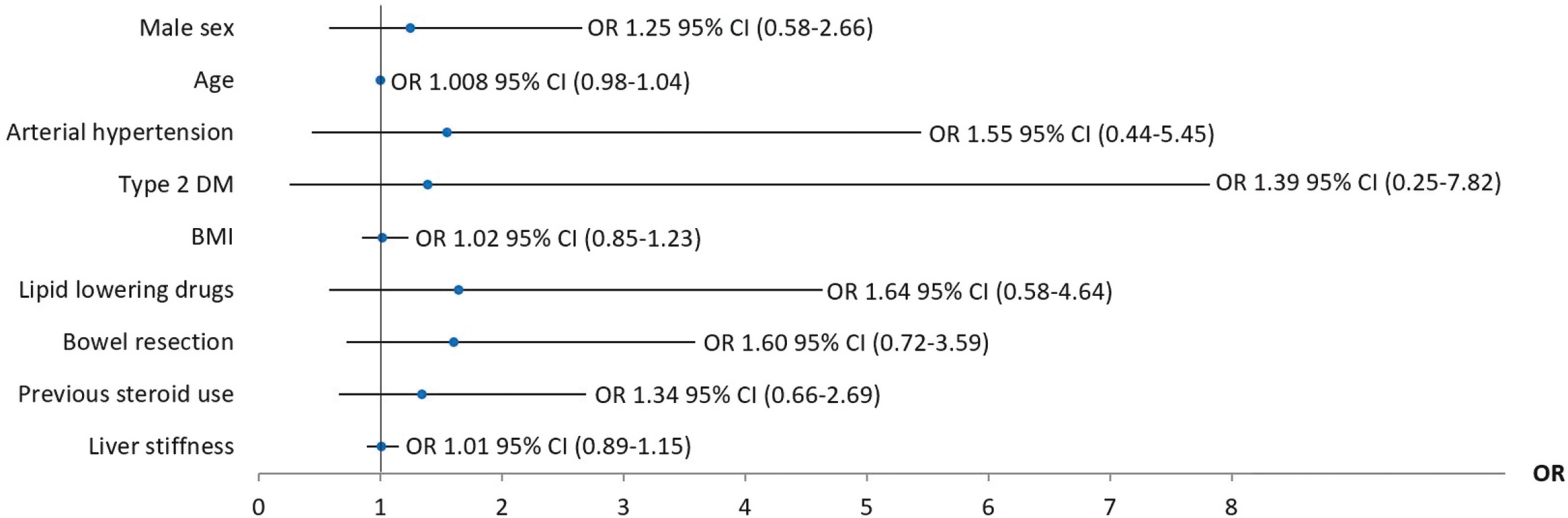
Logistic regression analysis of metabolic dysfunction–associated steatotic liver disease risk factors in the lean inflammatory bowel disease (IBD) group. Data are presented as odds ratio (OR) and 95% confidence interval (CI). Data were adjusted by sex, age, body mass index (BMI), waist-to-hip ratio, arterial hypertension, type 2 diabetes mellitus (DM), insulin resistance, smoking habit, lipid-lowering drugs use, and previous steroid use.

Univariable analysis identified male sex and older age as risk factors for MASLD in lean IBD participants. Moreover, the lean IBD group with MASLD had higher levels of liver stiffness (kPa), increased BMI levels, increased waist-hip ratio, worse glycemic control, increased insulin resistance, and poor lipid profile outcomes compared with the lean IBD group without MASLD (**Supplementary Table 3**). Nevertheless, these findings were not confirmed when multivariable analysis was performed (**Figure 5**). Although prior bowel resection was associated with MASLD on univariable analysis, no association was found between IBD related factors and MASLD on multivariable analysis.

### Comparison of Lean IBD Group and Overweight/Obese IBD Group With MASLD

The overweight/obese IBD group with MASLD showed significantly higher liver stiffness levels compared with the lean IBD group with MASLD (median 5.3 [range, 2.4-44.2] kPa vs 4.7 [range, 2.7-11.3] kPa; *P =* .007). In addition, arterial hypertension, prior smoking habit, poor glycemic control, and insulin resistance were more prevalent in the overweight/obese IBD group with MASLD. Noteworthy, no statistically differences were found in lipid profile except for lower high-density lipoprotein level in the overweight/obese IBD group with MASLD (median 49 [range, 25-101] mg/dL vs 57 [range, 34-133] mg/dL, *P =* .002)). Regarding IBD-related features, the overweight/obese IBD group with MASLD had older age at IBD diagnosis and lower prevalence of bowel resection compared with the lean IBD group with MASLD (**Supplementary Table 4**). In multivariable analysis, higher levels of the homeostatic model assessment of insulin resistance (OR, 1.49; 95% CI, 1.11-1.98; *P =* .007) and prior history of smoking (OR, 4.66; 95% CI, 1.17-18.49; *P =* .029) remained statistically significant. However, no IBD-related factors were statistically significant.

### Comparison of the Lean IBD Group With MASLD and the Lean Non-IBD Group With MASLD

We found no statistically significant differences between the lean IBD group with MASLD and the lean non-IBD group with MASLD in relation to anthropometric measures: BMI (22.8 ± 1.9 kg/m^2^ vs 23.6 ± 1.4 kg/m^2^; *P =* .259), body fat percentage (26.6 ± 6.9% vs 29.8 ± 6.0%; *P =* .217), and waist-to-hip ratio (0.90 ± 0.07 vs 0.89 ± 0.09; *P =* .719) In addition, no differences were observed in lipid profile or insulin resistance (**Supplementary Table 5**).

## Discussion

We present a detailed analysis of lean participants belonging to one of the largest IBD population cohorts to date assessing MASLD and liver fibrosis. To our knowledge, the evidence regarding MASLD and liver fibrosis in lean individuals with IBD is scarce. Adams et al^19^ published in 2018 a retrospective case-control study including 165 patients who previously underwent magnetic resonance imaging, most of them having an IBD diagnosis (around 80%). They concluded that underweight patients had a higher risk of MASLD compared with matched control subjects with normal weight.^19^ Moreover, Saroli Palumbo et al^20^ reported in 2018 an increased prevalence of MASLD and liver fibrosis in obese and overweight patients with IBD compared with lean patients with IBD. Both studies lacked a control group composed of lean participants without IBD. Subsequently, McHenry et al^21^ reported in 2019 a prevalence of MASLD of 20% in lean patients with CD and 10% in healthy lean control subjects, similar to our data. However, they did not assess associated risk factors and liver fibrosis in the lean population.^21^

High BMI has been recognized as an independent dose-dependent risk factor for MASLD. Consistently, our findings showed an increasing prevalence of MASLD with higher BMI levels for both the IBD and non-IBD groups. In addition, we found a MASLD prevalence of 10% in the lean non-IBD group from the general population, similar to the 5% to 10% reported by previous studies.^22-24^ Nevertheless, our key finding was the significantly higher prevalence of MASLD in the lean IBD group compared with the lean non-IBD group. Thus, we described that IBD is an independent risk factor for MASLD in lean individuals, after adjusting for classic metabolic risk factors and prior history of systemic steroid use.

Steroid treatment has been associated with weight gain and, consequently, with the development of liver steatosis. However, additional mechanisms have been described such as lipogenesis, gluconeogenesis, insulin resistance, and release of free fatty acids with subsequent uptake by the liver.^25^ Therefore, all patients with current or previous use of systemic steroid in the last 2 years were excluded from the analysis. Similarly, patients receiving methotrexate in the last 2 years were excluded due to its possible role in the development of MASLD.^26^

Beyond the epidemiological data, lean MASLD is a clinically relevant condition due to its close association with the components of metabolic syndrome compared with lean healthy population, leading to an increased all-cause and cardiovascular-related mortality.^27,28^ In addition, several IBD related risk factors for MASLD have been described such as longer IBD duration, disease activity, or older age at diagnosis of IBD.^29-31^ However, we found no association between IBD-related factors and MASLD development in lean IBD participants. In addition, we found no statistical differences when lean IBD participants with and without activity at inclusion were compared, probably due to insufficient statistical power related to the small proportion of patients with activity.

Noteworthy, the lean IBD group with MASLD had numerically lower BMI and body fat percentage compared with the lean non-IBD group with MASLD. On the one hand, this finding suggests that IBD could play a role in the development of MASLD at lower BMI. On the other hand, BMI probably does not fully reflect the risk of MASLD and metabolic disorders, so attention should be paid to other indicators such as waist-to-hip ratio, which was increased in the lean IBD group with MASLD compared with the lean non-IBD group. At the same time, participants in the lean IBD group with MASLD were more likely to have higher BMI levels and higher waist-to-hip ratio compared with those without MASLD, as in the general population.

We noticed an increased prevalence of arterial hypertension, poor glycemic control, and insulin resistance in the overweight/obese IBD group with MASLD compared with the lean IBD group with MASLD. These findings are biologically plausible, as obesity is a major driver of insulin resistance,^32^ and consistent with current evidence.^33^ Furthermore, we found no significant difference in serum cholesterol levels, as previously reported in general population based studies.^34^ Surprisingly, MASLD is associated with higher prevalence of metabolic syndrome, worse glycemic control, increased insulin resistance, and poor lipid profile outcomes in lean IBD participants, even in the absence of overweight or obesity. This highlights the importance of detecting and closely monitoring this group of patients.

Regarding significant liver fibrosis, the strongest predictor of specific mortality in MASLD,^35^ we found a numerically higher prevalence for the lean IBD group compared with the lean non-IBD group (4.7% vs 0%; *P* = 1.000). It is probable that we did not find statistically significant differences due to the small sample size of patients with significant liver fibrosis. This finding is similar to that of Rodriguez-Duque et al,^36^ who reported a 4-fold higher prevalence of advanced liver fibrosis in nonobese IBD patients with MASLD compared with nonobese patients without IBD. However, MASLD itself in the absence of liver cirrhosis plays an important role, as it has been described as a risk factor for atherosclerotic cardiovascular disease.

Our findings are consistent with the cumulative evidence in recent years on this topic. Thus, data coming from other immune-mediated conditions, such as psoriasis or hidradenitis suppurativa, also suggest that chronic inflammation could play a role in MASLD development.^37-39^ In parallel, the increased risk of MASLD in IBD participants independent of classic metabolic risk factors, such as obesity, supports this hypothesis. Therefore, our findings suggest that MASLD should also be suspected in lean patients with IBD, especially those with altered liver profile, and lifestyle measures could be prescribed to treat or prevent this entity. Future studies should be carried out to identify factors associated with MASLD development in IBD population to guide screening strategies, which should not be based only on BMI.

Finally, we acknowledge the following weaknesses of the study: (1) the lack of follow-up over time, inherent to the study design, (2) the small sample size of patients with significant liver fibrosis, (3) the lack of liver biopsies for histological confirmation of MASLD and detection of NASH, and (4) the absence of information on the cumulative dose of steroids received. Nevertheless, the main strengths of the study were its large sample size, the use of both US and TE to assess liver disease, and the presence of a comparable control group belonging to the same health area.

## Supplementary Material

izad175_suppl_Supplementary_Tables
