# Supplementary material for: Inflammatory Bowel Disease Is an Independent Risk Factor for Metabolic Dysfunction–Associated Steatotic Liver Disease in Lean Individuals
Source: Inflamm Bowel Dis. 2023 Aug 22;30(8):1274–83. doi: 10.1093/ibd/izad175 (PMC11291618; doi:10.1093/ibd/izad175)
Supplement: izad175_suppl_Supplementary_Tables [file izad175_suppl_supplementary_tables.docx]

SUPPLEMENTARY MATERIAL

***Supplementary Table 1***. Characteristics of lean participants according to IBD activity

| **Characteristics** | **Lean IBD with activity**  **(n= 13)** | **Lean IBD in remission**  **(n= 287)** | **Lean non-IBD**  **(n= 80)** | **Univariable**  **p value** | **Multivariable p value** |
| --- | --- | --- | --- | --- | --- |
| Female sex, n (%) | 7 (53.8) | 169 (58.9) | 46 (57.5) | *0.718  **0.805 |  |
| Age (years), mean ± SD | 43.1±15.6 | 46.9±13.0 | 44.1±15.5 | *0.298  **0.825 |  |
| BMI (Kg/m^2^), mean ± SD | 22.10±2.38 | 22.15±1.96 | 22.0±2.1 | *0.877  **0.970 |  |
| Waist-hip ratio, mean ± SD | 0.85±0.06 | 0.86±0.08 | 0.84±0.08 | *0.431  **0.896 |  |
| Arterial hypertension, n (%) | 1 (7.7) | 25 (8.7) | 8 (10.0) | *1.000  **1.000 |  |
| Type 2 DM, n (%) | 1 (7.7) | 11 (3.8) | 3 (3.8) | *0.418  **0.458 |  |
| Cardiovascular Disease, n (%) | 0 (0.0) | 6 (2.1) | - | *1.000 |  |
| Metabolic syndrome, n (%) | 1 (7.7) | 7 (2.4) | 1 (1.3) | *0.302  **0.261 |  |
| Chronic Kidney Disease, n (%) | 1 (7.7) | 22 (7.7) | 15 (18.8) | *1.000  **0.454 |  |
| MASLD, n (%) | 4 (30.8) | 60 (20.9) | 8 (10.0) | *0.486  **0.061 | **0.207 |
| Significant liver fibrosis in patients with MASLD, n (%) | 1 (25.0) | 2 (3.3) | 0 (0.0) | *0.179  **0.333 |  |
| Liver stiffness (kPa) in patients with MASLD, mean ± SD | 7.0±3.2 | 4.8±1.4 | 4.7± 0.5 | ***0.007**  **0.059 | ***0.011**  **OR 1.27 95%CI (1.06-1.52)** |
| Fasting glucose levels (mg/dl), median (range) | 95.0 (77.0-176.0) | 92.0 (67.0-299.0) | 93.0 (76.0-146.0) | *0.495  **0.814 |  |
| HbA1c (%), median (range) | 5.2 (4.7-6.3) | 5.4 (4.4-12.8) | 5.4 (4.2-8.9) | *0.151  **0.058 |  |
| Total cholesterol (mg/dl), mean ± SD | 161.9±37.5 | 191.3±40.1 | 197 ± 36.1 | ***0.010**  ****0.002** | *0.713  **0.158 |
| LDL (mg/dl), mean ± SD | 88.8±27.6 | 108.1±34.0 | 114.8± 34.3 | ***0.046**  ****0.012** | *0.852  **0.190 |
| HDL (mg/dl), mean ± SD | 54.9±13.7 | 64.5±17.8 | 63.5±14.0 | *0.056  ****0.046** | *0.082  **0.215 |
| Triglycerides (mg/dl), median (range) | 85 (47-659) | 80 (29-3087) | 81 (37-313) | *0.835  **0.768 |  |
| HOMA-IR, median (range) | 1.67 (0.38-0.79) | 1.12 (0.19-24.40) | 1.44 (0.39-10.50) | *0.116  **0.454 |  |

BMI: Body Mass Index. HbA1c: glycosylated haemoglobin. HDL: High Density Lipoproteins. HOMA: Homeostasis Model Assessment-Insulin Resistance. IBD: Inflammatory Bowel Disease. LDL: Low Density Lipoproteins. MASLD: Metabolic dysfunction-associated steatotic liver disease. Type 2 DM: Type 2 Diabetes Mellitus. For the qualitative variables Chi-square test or Fisher´s test were used, and for quantitative variables t-student or Mann-Whitney U test, as appropriate. Multivariable analysis included variables found to be significant in univariable analysis and biologically relevant variables such as age, sex, BMI, waist-to-hip ratio, arterial hypertension, type 2 DM, insulin resistance, smoking habit, lipid lowering drugs and previous steroid use. p-values in bold indicate statistical significance: p<0.05). *Lean IBD with activity vs Lean IBD in remission. **Lean IBD with activity vs lean non-IBD

***Supplementary Table 2***. Comparison of lean participants with MASLD and lean participants without MASLD

| **Characteristics** | **Lean participants (IBD and non-IBD) with MASLD (n= 72)** | **Lean participants (IBD and non-IBD) without MASLD**  **(n= 308)** | **p value*** |
| --- | --- | --- | --- |
| ***Clinical characteristics*** | | | |
| Male sex, n (%) | 41 (56.9) | 117 (38.0) | **0.003** |
| Age (years), mean ± SD | 50.8±13.5 | 45.2±13.5 | **0.002** |
| Active smokers, n (%) | 12 (16.7) | 66 (21.4) | 0.368 |
| Previous smokers, n (%) | 27 (37.5) | 116 (37.7) | 0.980 |
| Passive smokers, n (%) | 6 (8.3) | 37 (12.0) | 0.375 |
| Chronic Kidney Disease, n (%) | 6 (8.3) | 32 (10.4) | 0.601 |
| Cardiovascular Disease, n (%) | 2 (2.8) | 4 (1.3) | 0.319 |
| Cerebrovascular Disease, n (%) | 1 (1.4) | 3 (1.0) | 0.570 |
| Arterial hypertension, n (%) | 10 (13.9) | 24 (7.8) | 0.103 |
| Type 2 DM, n (%) | 5 (6.9) | 10 (3.2) | 0.147 |
| Hypercholesterolemia drugs, n (%) | 9 (12.5) | 29 (9.4) | 0.432 |
| Triglyceride lowering drugs, n (%) | 5 (6.9) | 7 (2.3) | **0.041** |
| Metabolic syndrome, n (%) | 5 (6.9) | 4 (1.3) | **0.015** |
| Liver Stiffness (kPa), median (range) | 4.7 (2.7-11.3) | 4.3 (2.1-30.5) | **0.005** |
| Significant liver fibrosis, n (%) | 3 (4.2) | 10 (3.2) | 0.719 |
| ***Anthropometric measures*** | | | |
| BMI (Kg/m^2^), median (range) | 23.3 (16.2-25.0) | 22.2 (15.4-25.0) | **<0.001** |
| Waist-hip ratio, mean ± SD | 0.90±0.07 | 0.85±0.07 | **<0.001** |
| Body fat percentage (%), median (range) CUN-BAE | 25.8 (6.4-39.7) | 27.8 (7.8-39.8) | 0.973 |
| Body fat percentage (%), mean ± SD BAI | 26.1±3.9 | 26.3±3.8 | 0.702 |
| ***Analytical characteristics*** | | | |
| Fasting glucose levels (mg/dl), median (range) | 95 (72-176) | 92 (67-299) | **0.013** |
| HbA1c (%), median (range) | 5.4 (4.6-7.3) | 5.4 (4.2-12.8) | 0.260 |
| Creatinine (mg/dl), median (range) | 0.83 (0.49-3.29) | 0.79 (0.37-4.10) | 0.262 |
| Serum albumin (g/dl), median (range) | 4.04 (3.16-4.70) | 4.04 (0.98-5.00) | 0.743 |
| Total cholesterol (mg/dl), mean ± SD | 190.0±40.3 | 192.0±39.5 | 0.768 |
| LDL (mg/dl), mean ± SD | 108.0±34.4 | 109.0±34.1 | 0.907 |
| HDL (mg/dl), mean ± SD | 58.3±17.4 | 65.3±16.7 | **0.002** |
| Triglycerides (mg/dl), median (range) | 110 (37-659) | 76 (29-3087) | **<0.001** |
| C-reactive protein (mg/L), median (range) | 2.01 (0.03-219.00) | 1.50 (0.00-37.40) | 0.117 |
| Fasting insulin levels (µU/ml), median (range) | 6.7 (2.0-35.6) | 4.8 (2.0-65.0) | **0.002** |
| HOMA-IR, median (range) | 1.6 (0.4-10.3) | 1.1 (0.2-24.4) | **<0.001** |
| ***IBD-related features*** | | | |
| IBD, n (%) | 64 (88.9) | 236 (76.6) | **0.022** |
| ***Type of IBD, n (%)*** |  |  |  |
| UC | 30 (41.7) | 127 (41.2) | 0.946 |
| CD | 31 (43.1) | 106 (34.4) | 0.169 |
| IC | 3 (4.2) | 3 (1.0) | 0.085 |

BAI: Body Adiposity Index. BMI: Body Mass Index. CD: Crohn´s Disease. CUN-BAE: Clínica Universidad de Navarra-Body Adiposity Estimator. HbA1c: glycosylated haemoglobin. HDL: High Density Lipoproteins. HOMA: Homeostasis Model Assessment-Insulin Resistance. IBD: Inflammatory Bowel Disease. Lean: BMI < 25 kg/m^2^. MASLD: Metabolic dysfunction-associated steatotic liver disease. Non-lean (Overweight/Obese): BMI ≥25 kg/m^2^. LDL: Low Density Lipoproteins. SD: Standard Deviation. Type 2 DM: Type 2 Diabetes Mellitus. UC: Ulcerative Colitis *For the qualitative variables Chi-square test or Fisher´s test were used, and for quantitative variables t-student or Mann-Whitney U test, as appropriate (p values in bold indicate statistical significance: p<0.05)

***Supplementary Table 3***. Comparison of lean IBD group according to MASLD diagnosis

| **Characteristics** | **Lean IBD with MASLD (n= 64)** | **Lean IBD without MASLD (n= 236)** | **p value*** |
| --- | --- | --- | --- |
| ***Clinical characteristics*** | | | |
| Male sex, n (%) | 37 (57.8) | 87 (36.9) | **0.003** |
| Age (years), mean ± SD | 50.1±13.4 | 45.9±12.9 | **0.024** |
| Active smokers, n (%) | 12 (18.8) | 51 (21.6) | 0.618 |
| Previous smokers, n (%) | 23 (35.9) | 98 (41.5) | 0.419 |
| Passive smokers, n (%) | 6 (9.4) | 27 (11.4) | 0.639 |
| Chronic Kidney Disease, n (%) | 3 (4.7) | 20 (8.5) | 0.430 |
| Cardiovascular Disease, n (%) | 2 (3.1) | 4 (1.7) | 0.611 |
| Cerebrovascular Disease, n (%) | 1 (1.6) | 1 (0.4) | 0.382 |
| Arterial hypertension, n (%) | 7 (10.9) | 19 (8.1) | 0.467 |
| Type 2 DM, n (%) | 4 (6.3) | 8 (3.4) | 0.292 |
| Hypercholesterolemia drugs, n (%) | 9 (14.1) | 26 (11.0) | 0.501 |
| Triglyceride lowering drugs, n (%) | 4 (6.3) | 6 (2.5) | 0.229 |
| Metabolic syndrome, n (%) | 4 (6.3) | 4 (1.7) | 0.067 |
| Liver Stiffness (kPa), median (range) | 4.7 (2.7-11.3) | 4.3 (2.1-30.5) | **0.012** |
| Significant liver fibrosis, n (%) | 3 (4.7) | 10 (4.2) | 1.000 |
| ***Anthropometric measures*** | | | |
| BMI (Kg/m^2^), mean ± SD | 22.8±1.9 | 22.0±1.9 | **0.004** |
| Waist-hip ratio, mean ± SD | 0.90±0.07 | 0.85±0.07 | **<0.001** |
| Body fat percentage (%), mean ± SD CUN-BAE | 26.6±6.9 | 27.4±6.5 | 0.430 |
| Body fat percentage (%), mean ± SD BAI | 25.9±3.9 | 26.2±3.7 | 0.482 |
| ***Analytical characteristics*** | | | |
| Fasting glucose levels (mg/dl), median (range) | 95.5 (72.0-176.0) | 92.0 (67.0-299.0) | **0.006** |
| HbA1c (%), median (range) | 5.4 (4.6-7.3) | 5.4 (4.4-12.8) | 0.194 |
| Creatinine (mg/dl), median (range) | 0.83 (0.49-3.29) | 0.79 (0.37-4.10) | 0.299 |
| Serum albumin (g/dl), median (range) | 4.05 (3.16-4.70) | 4.04 (0.98-5.00) | 0.730 |
| Total cholesterol (mg/dl), mean ± SD | 188.0±40.9 | 191.0±40.3 | 0.710 |
| LDL (mg/dl), mean ± SD | 107.0±33.9 | 107.0±34.0 | 0.859 |
| HDL (mg/dl), mean ± SD | 58.3±17.7 | 65.7±17.4 | **0.003** |
| Triglycerides (mg/dl), median (range) | 110 (37-659) | 77 (29-3087) | **<0.001** |
| C-reactive protein (mg/L), median (range) | 2.10 (0.03-219.00) | 1.71 (0.02-37.40) | 0.288 |
| Fasting insulin levels (µU/ml), median (range) | 6.6 (2.0-35.6) | 4.5 (2.0-65.0) | **0.002** |
| HOMA-IR, median (range) | 1.6 (0.4-10.3) | 1.0 (0.2-24.4) | **<0.001** |
| ***IBD-related features*** | | | |
| Age at diagnosis (years), median (range) | 32.0 (12.0-65.0) | 31.5 (9.0-71.0) | 0.226 |
| IBD duration (years), median (range) | 12.0 (0.0-38.0) | 11.0 (0.0-46.0) | 0.211 |
| ***Type of IBD, n (%)*** |  |  |  |
| UC | 30 (46.9) | 127 (53.8) | 0.324 |
| CD | 31 (48.4) | 106 (44.9) | 0.616 |
| IC | 3 (4.7) | 3 (1.3) | 0.114 |
| ***Extent of UC, n (%)*** (n= 157) |  |  |  |
| Proctitis | 8 826.7) | 33 (26.0) | 0.939 |
| Left-sided colitis | 8 (26.7) | 45 (35.4) | 0.361 |
| Extensive colitis | 14 (46.7) | 49 (38.6) | 0.417 |
| ***Extent of CD, n (%)*** (n= 137) |  |  |  |
| Ilecolonic (L3) | 15 (48.4) | 49 (46.2) | 0.832 |
| Terminal ileum (L1) | 11 (35.5) | 36 (34.0) | 0.875 |
| Colonic (L2) | 3 (9.7) | 12 (11.3) | 1.000 |
| Terminal ileum (L1) + L4 | 1 (3.2) | 1 (0.9) | 0.403 |
| Ileocolonic (L3) + L4 | 0 (0.0) | 4 (3.8) | 0.574 |
| Isolated upper disease (L4) | 1 (3.2) | 3 (2.8) | 1.000 |
| Colonic (L2) + L4 | 0 (0.0) | 1 (0.9) | 1.000 |
| ***CD behaviour, n (%)*** (n= 137) |  |  |  |
| Inflammatory | 21 (67.7) | 66 (62.3) | 0.577 |
| Stricturing | 5 (16.1) | 30 (28.3) | 0.172 |
| Penetrating | 5 (16.1) | 9 (8.5) | 0.217 |
| Stricturing + Penetrating | 0 (0.0) | 1 (0.9) | 1.000 |
| ***Previous surgery, n (%)*** |  |  |  |
| Bowel resection | 16 (25.0) | 31 (13.1) | **0.021** |
| Perianal | 2 (3.1) | 11 (4.7) | 0.742 |
|  |  |  |  |
| Extraintestinal manifestations, n (%) | 11 (17.2) | 53 (22.5) | 0.361 |
| Perianal disease, n (%) | 8 (12.5) | 32 (13.6) | 0.825 |
| IBDQ-9, median (range) | 51.0 (29.0-59.0) | 51.0 (27.0-58.0) | 0.426 |
| ***Clinical severity,*** n (%) |  |  |  |
| **Partial Mayo score**  (n= 157)  Remission  Active disease | 29 (96.7)  1 (3.3) | 122 (96.1)  5 (3.9) | 1.000 |
| **Harvey-Bradshaw index**  (n= 137)  Remission  Active disease | 28 (90.3)  3 (9.7) | 102 (96.2)  4 (3.8) | 0.191 |
| ***Current treatment,*** n (%) |  |  |  |
| Only 5-ASA | 22 (34.4) | 85 (36.0) | 0.808 |
| Anti-TNF | 13 (20.3) | 52 (22.0) | 0.767 |
| Only Thiopurines | 7 (10.9) | 22 (9.3) | 0.698 |
| Ustekinumab | 5 (7.8) | 12 (5.1) | 0.403 |
| Biologic therapy + Thiopurines | 1 (1.6) | 14 (5.9) | 0.207 |
| Vedolizumab | 0 (0.0) | 8 (3.4) | 0.210 |
| ***Previous treatment, n (%)*** |  |  |  |
| Systemic steroids since diagnosis | 45 (70.3) | 151 (64.3) | 0.366 |
| Systemic steroids last 5 years | 19 (29.7) | 63 (26.8) | 0.647 |
| Other biologic agent | 16 (25.0) | 46 (19.6) | 0.343 |

BAI: Body Adiposity Index. BMI: Body Mass Index. CD: Crohn´s Disease. CUN-BAE: Clínica Universidad de Navarra-Body Adiposity Estimator. HbA1c: glycosylated haemoglobin. HDL: High Density Lipoproteins. HOMA: Homeostasis Model Assessment-Insulin Resistance. IBD: Inflammatory Bowel Disease. Lean: BMI < 25 kg/m^2^. MASLD: Metabolic dysfunction-associated steatotic liver disease. Non-lean (Overweight/Obese): BMI ≥25 kg/m^2^. LDL: Low Density Lipoproteins. SD: Standard Deviation. Type 2 DM: Type 2 Diabetes Mellitus. UC: Ulcerative Colitis *For the qualitative variables Chi-square test or Fisher´s test were used, and for quantitative variables t-student or Mann-Whitney U test, as appropriate (p values in bold indicate statistical significance: p<0.05)

***Supplementary Table 4***. Comparison of lean IBD group with MASLD and overweight/obese IBD group with MASLD

| **Characteristics** | **Lean IBD with MASLD (n= 64)** | **Overweight/obese IBD with MASLD (n= 231)** | **p value*** |
| --- | --- | --- | --- |
| ***Clinical characteristics*** | | | |
| Male sex, n (%) | 37 (57.8) | 143 (61.9) | 0.553 |
| Age (years), mean ± SD | 50.1±13.4 | 54.1±12.6 | **0.027** |
| Active smokers, n (%) | 12 (18.8) | 40 (17.3) | 0.790 |
| Previous smokers, n (%) | 23 (35.9) | 124 (53.7) | **0.012** |
| Passive smokers, n (%) | 6 (9.4) | 40 (17.3) | 0.121 |
| Chronic Kidney Disease, n (%) | 3 (4.7) | 30 (13.0) | 0.073 |
| Cardiovascular Disease, n (%) | 2 (3.1) | 16 (6.9) | 0.261 |
| Cerebrovascular Disease, n (%) | 1 (1.6) | 4 (1.7) | 1.000 |
| Arterial hypertension, n (%) | 7 (10.9) | 65 (28.1) | **0.005** |
| Type 2 DM, n (%) | 4 (6.3) | 26 (11.3) | 0.349 |
| Hypercholesterolemia drugs, n (%) | 9 (14.1) | 58 (25.1) | 0.062 |
| Triglyceride lowering drugs, n (%) | 4 (6.3) | 30 (13.0) | 0.184 |
| Liver Stiffness (kPa), median (range) | 4.7 (2.7-11.3) | 5.3 (2.4-44.2) | **0.007** |
| Significant liver fibrosis, n (%) | 3 (4.7) | 28 (12.1) | 0.107 |
| ***Anthropometric measures*** | | | |
| Waist-hip ratio, mean ± SD | 0.90±0.07 | 0.96±0.08 | **<0.001** |
| Body fat percentage (%), median (range) CUN-BAE | 25.6 (6.4-39.7) | 34.4 (22.9-52.7) | **<0.001** |
| Body fat percentage (%), median (range) BAI | 25.4 (18.4-34.4) | 31.3 (21.8-53.4) | **<0.001** |
| ***Analytical characteristics*** | | | |
| Fasting glucose levels (mg/dl), median (range) | 95.5 (72.0-176.0) | 102.0 (65.0-431.0) | **0.007** |
| HbA1c (%), median (range) | 5.4 (4.6-7.3) | 5.6 (4.1-8.2) | **0.010** |
| Creatinine (mg/dl), median (range) | 0.83 (0.49-3.29) | 0.86 (0.30-3.66) | 0.213 |
| Serum albumin (g/dl), mean ± SD | 4.03±0.35 | 4.03±0.33 | 0.865 |
| Total cholesterol (mg/dl), mean ± SD | 188.0±40.9 | 191.0±36.7 | 0.627 |
| LDL (mg/dl), mean ± SD | 107.0±33.9 | 113.0±33.8 | 0.153 |
| HDL (mg/dl), median (range) | 57 (34-133) | 49 (25-101) | **0.002** |
| Triglycerides (mg/dl), median (range) | 110 (37-659) | 120 (30-678) | 0.259 |
| Total bilirubin (mg/dl), median (range) | 0.51 (0.13-1.82) | 0.50 (0.10-3.66) | 0.790 |
| AST (UI/L), median (range) | 21.5 (10.0-57.0) | 21.0 (10.0-137.0) | 0.708 |
| ALT (UI/L), median (range) | 19.0 (5.0-52.0) | 20.0 (6.0-96.0) | 0.228 |
| GGT (UI/L), median (range) | 22.5 (9.0-73.0) | 27.0 (9.0-1372.0) | 0.107 |
| ALP (UI/L), median (range) | 73.0 (32.0-138.0) | 72.0 (20.0-164.0) | 0.505 |
| Platelet count (10^3^/mm^3^), mean ± SD | 267±71 | 256±63 | 0.223 |
| C-reactive protein (mg/L), median (range) | 2.10 (0.03-219.00) | 3.10 (0.10-159.00) | 0.018 |
| Fasting insulin levels (µU/ml), median (range) | 6.6 (2.0-35.6) | 12.0 (2.0-130.0) | **<0.001** |
| HOMA-IR, median (range) | 1.61 (0.38-10.30) | 2.98 (0.32-45.90) | **<0.001** |
| ***IBD-related features*** | | | |
| Age at diagnosis (years), median (range) | 32 (12-65) | 37 (11-74) | **0.021** |
| IBD duration (years), median (range) | 12 (0-38) | 13 (0-42) | 0.925 |
| ***Type of IBD, n (%)*** |  |  |  |
| UC | 30 (46.9) | 129 (55.8) | 0.203 |
| CD | 31 (48.4) | 100 (43.3) | 0.463 |
| IC | 3 (4.7) | 2 (0.9) | 0.070 |
| ***Extent of UC, n (%)*** (n= 159) |  |  |  |
| Proctitis | 8 (26.7) | 35 (27.1) | 0.959 |
| Left-sided colitis | 8 (26.7) | 47 (36.4) | 0.311 |
| Extensive colitis | 14 (46.7) | 47 (36.4) | 0.299 |
| ***Extent of CD, n (%)*** (n= 131) |  |  |  |
| Ilecolonic (L3) | 15 (48.4) | 45 (45.0) | 0.741 |
| Terminal ileum (L1) | 11 (35.5) | 34 (34.0) | 0.879 |
| Colonic (L2) | 3 (9.7) | 13 (13.0) | 0.761 |
| Terminal ileum (L1) + L4 | 1 (3.2) | 4 (4.0) | 1.000 |
| Ileocolonic (L3) + L4 | 0 (0.0) | 3 (3.0) | 1.000 |
| Isolated upper disease (L4) | 1 (3.2) | 0 (0.0) | 0.237 |
| Colonic (L2) + L4 | 0 (0.0) | 1 (1.0) | 1.000 |
| ***CD behaviour, n (%)*** (n= 131) |  |  |  |
| Inflammatory | 21 (67.7) | 50 (50.0) | 0083 |
| Stricturing | 5 (16.1) | 35 (35.0) | **0.046** |
| Penetrating | 5 (16.1) | 12 (12.0) | 0.550 |
| Stricturing + Penetrating | 0 (0.0) | 3 (3.0) | 1.000 |
| ***Previous surgery, n (%)*** |  |  |  |
| Bowel resection | 16 (25.0) | 30 (13.0) | **0.019** |
| Perianal | 2 (3.1) | 22 (9.5) | 0.123 |
|  |  |  |  |
| Extraintestinal manifestations, n (%) | 11 (17.2) | 43 (18.6) | 0.794 |
| Perianal disease, n (%) | 8 (12.5) | 27 (11.7) | 0.859 |
| IBDQ-9, median (range) | 51 (29-59) | 51 (25-59) | 0.782 |
| ***Clinical severity,*** n (%) |  |  |  |
| **Partial Mayo score**  (n= 159)  Remission  Active disease | 29 (96.7)  1 (3.3) | 121 (93.8)  8 (6.2) | 1.000 |
| **Harvey-Bradshaw index**  (n= 131)  Remission  Active disease | 28 (90.3)  3 (9.7) | 96 (96.0)  4 (4.0) | 0.355 |
| ***Current treatment,*** n (%) |  |  |  |
| Only 5-ASA | 22 (34.4) | 89 (38.5) | 0.544 |
| Anti-TNF | 13 (20.3) | 44 (19.0) | 0.821 |
| Only Thiopurines | 7 (10.9) | 30 (13.0) | 0.661 |
| Ustekinumab | 5 (7.8) | 10 (4.3) | 0.262 |
| Biologic therapy + Thiopurines | 1 (1.6) | 14 (6.1) | 0.205 |
| Vedolizumab | 0 (0.0) | 12 (5.2) | 0.076 |
| ***Previous treatment, n (%)*** |  |  |  |
| Systemic steroids since diagnosis | 45 (70.3) | 151 (65.7) | 0.484 |
| Systemic steroids last 5 years | 19 (29.7) | 63 (27.3) | 0.703 |
| Other biologic agent | 16 (25.0) | 45 (19.5) | 0.335 |

ALP: Alkaline Phosphatase. ALT: Alanine Aminotransferase. AST: Aspartate Aminotrasferase. BAI: Body Adiposity Index. BMI: Body Mass Index. CD: Crohn´s Disease. CUN-BAE: Clínica Universidad de Navarra-Body Adiposity Estimator. GGT: Gamma-Glutamyl Transferase. HbA1c: glycosylated haemoglobin. HDL: High Density Lipoproteins. HOMA: Homeostasis Model Assessment-Insulin Resistance. IBD: Inflammatory Bowel Disease. Lean: BMI < 25 kg/m^2^. MASLD: Metabolic dysfunction-associated steatotic liver disease. Non-lean (Overweight/Obese): BMI ≥25 kg/m^2^. LDL: Low Density Lipoproteins. SD: Standard Deviation. Type 2 DM: Type 2 Diabetes Mellitus. UC: Ulcerative Colitis *For the qualitative variables Chi-square test or Fisher´s test were used, and for quantitative variables t-student or Mann-Whitney U test, as appropriate (p values in bold indicate statistical significance: p<0.05)

***Supplementary Table 5***. Comparison of lean IBD group with MASLD and lean non-IBD group with MASLD

| **Characteristics** | **Lean IBD with MASLD (n= 64)** | **Lean non-IBD with MASLD**  **(n= 8)** | **p value*** |
| --- | --- | --- | --- |
| ***Clinical characteristics*** | | | |
| Male sex, n (%) | 37 (57.8) | 4 (50.0) | 0.719 |
| Age, mean ± SD | 50.1±13.4 | 56.1 ± 13.9 | 0.236 |
| Active smokers, n (%) | 12 (18.8) | 0 (0.0) | 0.337 |
| Previous smokers, n (%) | 23 (35.9) | 4 (50.0) | 0.463 |
| Passive smokers, n (%) | 6 (9.4) | 0 (0.0) | 1.000 |
| Chronic Kidney Disease, n (%) | 3 (4.7) | 3 (37.5) | **0.016** |
| Cardiovascular Disease, n (%) | 2 (3.1) | 0 (0.0) | 1.000 |
| Cerebrovascular Disease, n (%) | 1 (1.6) | 0 (0.0) | 1.000 |
| Arterial hypertension, n (%) | 7 (10.9) | 3 (37.5) | 0.075 |
| Type 2 DM, n (%) | 4 (6.3) | 1 (12.5) | 0.455 |
| Hypercholesterolemia drugs, n (%) | 9 (14.1) | 0 (0.0) | 0.584 |
| Triglyceride lowering drugs, n (%) | 4 (6.3) | 1 (12.5) | 0.455 |
| Liver Stiffness (kPa), mean ± SD | 4.9±1.6 | 4.7 ± 0.4 | 0.632 |
| Significant liver fibrosis, n (%) | 3 (4.7) | 0 (0.0) | 1.000 |
| ***Anthropometric measures*** | | | |
| BMI (Kg/m^2^), mean ± SD | 22.8±1.9 | 23.6 ± 1.4 | 0.259 |
| Waist-hip ratio, mean ± SD | 0.90±0.07 | 0.89 ± 0.09 | 0.719 |
| Body fat percentage (%), mean ± SD CUN-BAE | 26.6±6.9 | 29.8 ± 6.0 | 0.217 |
| Body fat percentage (%), mean ± SD BAI | 25.9±3.9 | 27.9 ± 4.5 | 0.187 |
| ***Analytical characteristics*** | | | |
| Fasting glucose levels (mg/dl), median (range) | 95.5 (72.0-176.0) | 95.0 (76.0-100.0) | 0.481 |
| HbA1c (%), mean ± SD | 5.5±0.5 | 5.4 ± 0.4 | 0.695 |
| Creatinine (mg/dl), median (range) | 0.83 (0.49-3.29) | 0.91 (0.62-1.02) | 0.582 |
| Serum albumin (g/dl), mean ± SD | 4.0±0.3 | 4.1 ± 0.2 | 0.610 |
| Total cholesterol (mg/dl), mean ± SD | 188.0±40.9 | 207.0± 32.0 | 0.252 |
| LDL (mg/dl), mean ± SD | 107.0±33.9 | 124.0±37.9 | 0.200 |
| HDL (mg/dl), mean ± SD | 58.3±17.7 | 59.0 ± 15.8 | 0.915 |
| Triglycerides (mg/dl), median (range) | 110 (37-659) | 96 (79-224) | 0.728 |
| C-reactive protein (mg/L), median (range) | 2.10 (0.03-219.00) | 1.13 (0.54-3.18) | 0.207 |
| Fasting insulin levels (µU/ml), median (range) | 6.55 (2.00-35.60) | 7.07 (4.34-17.50) | 0.631 |
| HOMA-IR, median (range) | 1.61 (0.38-10.30) | 1.71 (0.81-4.15) | 0.877 |

BAI: Body Adiposity Index. BMI: Body Mass Index. CUN-BAE: Clínica Universidad de Navarra-Body Adiposity Estimator. HbA1c: glycosylated haemoglobin. HDL: High Density Lipoproteins. HOMA: Homeostasis Model Assessment-Insulin Resistance. IBD: Inflammatory Bowel Disease. MASLD: Metabolic dysfunction-associated steatotic liver disease. LDL: Low Density Lipoproteins. SD: Standard Deviation. Type 2 DM: Type 2 Diabetes Mellitus. *For the qualitative variables Chi-square test or Fisher´s test were used, and for quantitative variables t-student or Mann-Whitney U test, as appropriate (p values in bold indicate statistical significance: p<0.05)
